# Supplementary material for: A Fruit-Expressed MYB Transcription Factor Regulates Anthocyanin Biosynthesis in Atropa belladonna
Source: Int J Mol Sci. 2024 May 2;25(9):4963. doi: 10.3390/ijms25094963 (PMC11084770; doi:10.3390/ijms25094963)
Supplement: Supplementary file 1 [file ijms-25-04963-s001.zip › ijms-2974900-supplementary.pdf]

```
1                         cggccgggggactttacctcacgtgctttgcctgaactcaatgattggagttctctttttccattttggcgtc
96    ATGAATTCTACTAGTAGTACTAAATCATCAGAGGTCAGGAAAGGTGCATGGACCGAAGAAGAAGATCTACTTTTGAGAAAATGTATTGAC
       M  N  S  T  S  S  T  K  S  S  E  V  R  K  G  A  W  T  E  E  E  D  L  L  L  R  K  C  I  D
186   AAGTACGGTGAAGGAAAGTGGCATCAAATTCCCATTAGAGCTGGTCTAAATAGATGCAGAAAGAGCTGCAGACTGAGGTGGTTGAATTAT
       K  Y  G  E  G  K  W  H  Q  I  P  I  R  A  G  L  N  R  C  R  K  S  C  R  L  R  W  L  N  Y
276   CTAAGGCCACATATTAAGAGAGGTGACTTCGCTTCGGATGAAATAGATCTCATCTTCAGGCTTCATAAGCTCTTAGGCAACAGATGGTCA
       L  R  P  H  I  K  R  G  D  F  A  S  D  E  I  D  L  I  F  R  L  H  K  L  L  G  N  R  W  S
366   CTTATTGCTGGTAGACTTCCGGGAAGAACAGCAAACGATGTGAAAAATTACTGGAACACACACCTACATAGAAAGTTAATTTCTCCTCAG
       L  I  A  G  R  L  P  G  R  T  A  N  D  V  K  N  Y  W  N  T  H  L  H  R  K  L  I  S  P  Q
456   AGACAGGAGAGAAAGTACAATAATGCCCTCAAGATCACTGAAAACACCATAATAAGACCTTGGCCTCGGACCTTCTCAAGTGCAACGAAA
       R  Q  E  R  K  Y  N  N  A  L  K  I  T  E  N  T  I  I  R  P  W  P  R  T  F  S  S  A  T  K
546   CCTTGGTGCAACAACAAAAGTATCACAAATACAATAGACAAAGACAAGCATCGCAACAACGAAACAGTACTAAATATTTGTGAGAAGCCA
       P  W  C  N  N  K  S  I  T  N  T  I  D  K  D  K  H  R  N  N  E  T  V  L  N  I  C  E  K  P
636   ACAGGCGAAACAACGAGGTCGTCGATGGACGAGGGAGTTCAATGGTGGACAAGTTTACTGGAAAATTGCAATGAATTTGAGGAAGCAGTT
       T  G  E  T  T  R  S  S  M  D  E  G  V  Q  W  W  T  S  L  L  E  N  C  N  E  F  E  E  A  V
726   GGGAGATTTGATGAAGAAAATAAGTTACCAAGTTTGTTGCATGACGAAAACTCACCGCCAATGCAAGAAGGACAAAGTGATGGCTGGAAT
       G  R  F  D  E  E  N  K  L  P  S  L  L  H  D  E  N  S  P  P  M  Q  E  G  Q  S  D  G  W  N
816   GACTTTTCTGCTGATATTGACCTATGGGATCTACTTAATTAAttagtttcatcaatcaccattaaacacatagtataattaatggtattg
       D  F  S  A  D  I  D  L  W  D  L  L  N  *
906   tgtacttgtaatagggatacattctattctatgccgtttgaactttgtatcccaagtacaagtagggatctattactattttcattctag
996   agtttaatttataattataccgtggaaaaaattttacatc
```

Figure S1. The cDNA sequence and derived amino acids sequence of *AbMYB1*.

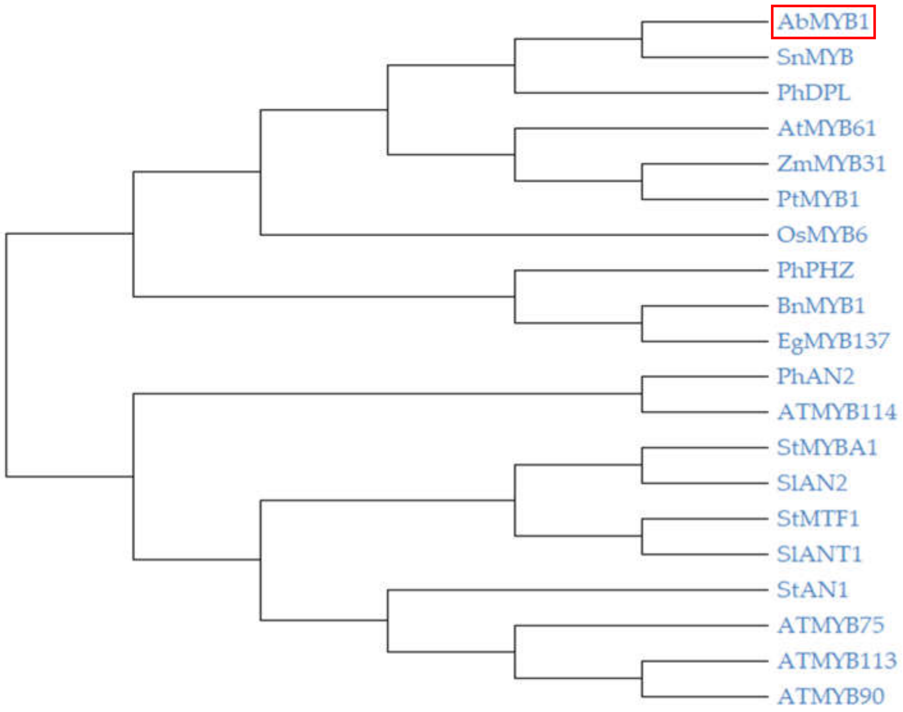

Figure S2. The Phylogenetic Relationships of AbMYB1 with Other R2R3-MYBs. *Solanum nigrum (Sn), Petunia hybrid (Ph), Zea mays (Zm), Pinus taeda (Pt), Oryza sativa (Os), Boehmeria nivea (Bn), Eucalyptus gunnii (Eg), Solanum tuberosum (St), Solanum lycopersicum (Sl), Arabidopsis thaliana (At).* SnMYB (AVT28142.1), PhDPL (ADQ00394.1), ZmMYB31 (NP_001105949.2), PtMYB1 (AAQ62541.1), AtMYB61 (AT1G09540), OsMYB6 (QBM91277.1), BnMYB1 (AXF84304.1), PhPHZ (ADW94951.1), EgMYB137 (APX44023.1), PhAN2 (AUH28246.1), StMYBA1 (ALA13582.1), SlAN2 (NP_001265992.1), StMTF1 (EU310399), SlANT1 (NP_001234417.1), StAN1 (AAX53093.1), ATMYB75 (AT1G56650.1), ATMYB114 (AT1G66380.1), ATMYB113 (AT1G66370.1), ATMYB90 (AT1G66390.1).

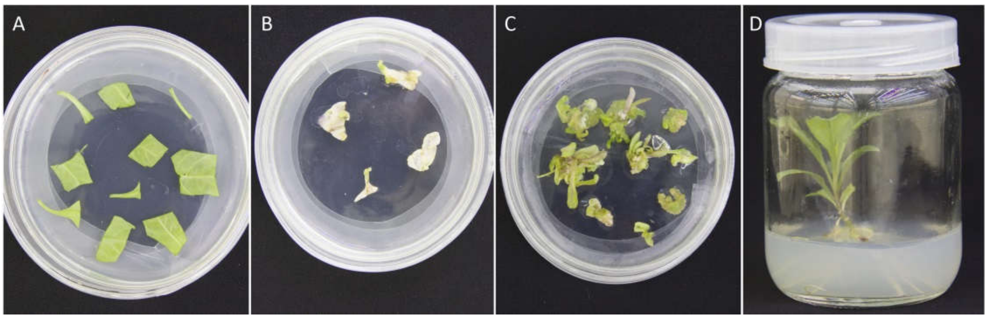

Figure S3. The process of agrobacterium-mediated genetic transformation.（A）, Leaf discs co-cultured with agrobacterium; （B） Resistant callus; （C） Resistant buds on rooting medium; （D） Regenerated plants.

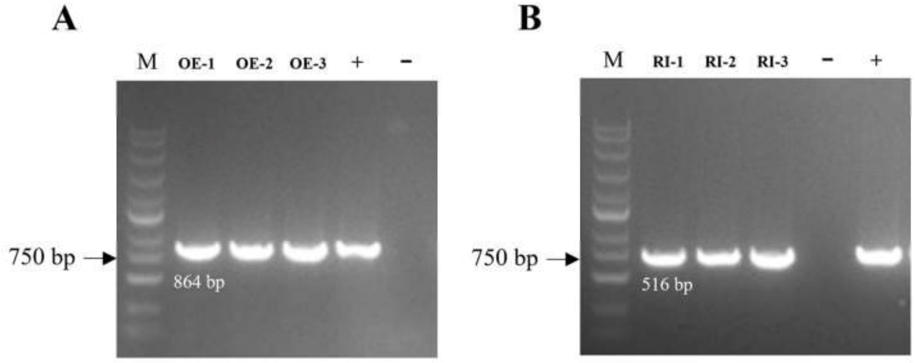

Figure S4. Detection of transgenic plants by PCR. (A) Target gene detection in *AbMYB1*-overexpression plants. (B) Target gene detection in *AbMYB1*-RNAi plants. M, Marker (15000 bp-100 bp); +, Positive control (recombinant plasmid containing the target gene); -: Negative control (wild type *A. belladonna*); OE-1, OE-2 and OE-3: the *AbMYB1*-overexpression transgenic plant lines; RI-1, RI-2 and RI-3: the *AbMYB1*-RNAi transgenic pant lines.

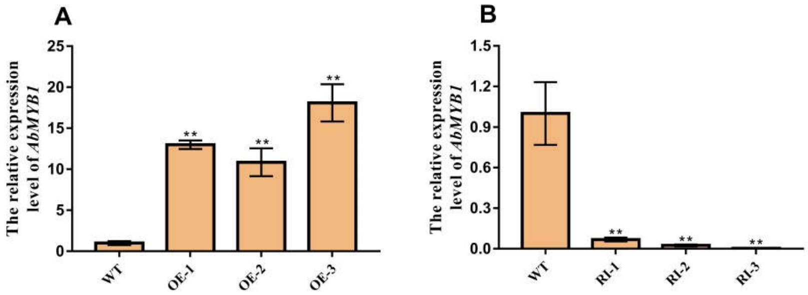

Figure S5. The *AbMYB1* expression levels in the flowers of transgenic lines. (A) Relative expression of *AbMYB1* in *AbMYB1*-overexpression plants. (B) Relative expression of *AbMYB1* in *AbMYB1* -RNAi lines. WT, wild type *A. belladonna*; *OE-1*, *OE-2* and *OE-3*: the *AbMYB1*-overexpression transgenic plant lines; *RI-1*, *RI-2* and *RI-3*: the *AbMYB1* -RNAi transgenic pant lines; ** means significant difference in T test (P< 0.01).

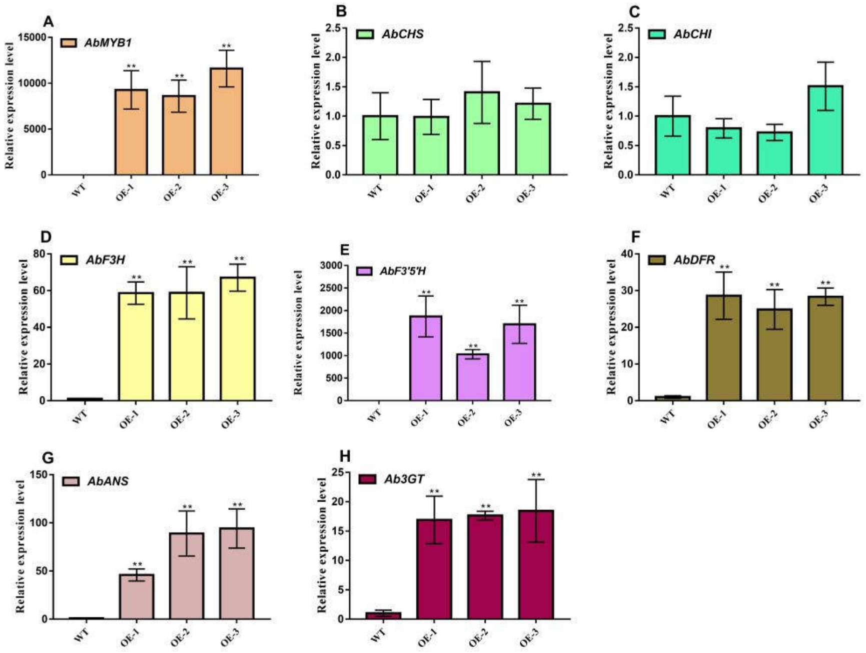

Figure S6. The relative expression of genes related to anthocyanin biosynthesis in *A. belladonna* leaves. A, the expression level of *AbMYB1* in different lines; B, the expression level of *AbCHS* in different lines; C, the expression level of *AbCHI* in different lines; D, the expression level of *AbF3H* in different lines; E, the expression level of *AbF3′5′H* in different lines; F, the expression level of *AbDFR* in different lines; G, the expression level of *AbANS* in different lines; H, the expression level of *Ab3GT* in different lines; WT, the wild type plants; *OE-1*, *OE-2* and *OE-3*: the *AbMYB1*-overexpression transgenic plant lines; ** means significant difference in T test (P< 0.01).

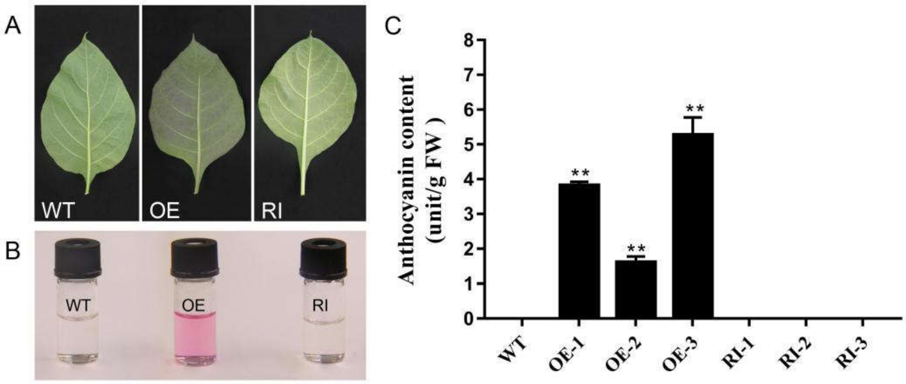

Figure S7. The anthocyanin accumulation in the leaves of transgenic *A. belladonna* plants. (A) the anthocyanin accumulation in the leaves of transgenic plant lines and wild type plant lines; (B) total anthocyanin extracted from the leaves of transgenic plant lines and wild type plant lines; (C) total anthocyanin content in the leaves of transgenic plant lines and wild type plant lines. WT, the leaves of wild type plants; *OE-1*, *OE-2* and *OE-3*: the leaves of *AbMYB1*-overexpression plants; *RI-1*, *RI-2* and *RI-3*: the leaves of *AbMYB1*-RNAi plants; ** means significant difference in T test ($P< 0.01$).

Table S1. Primers used in PCR experiments.

Primers	Sequences（5'-3'）	Application
F-cAbMYB1	CGGCCGGGGGACTTTACCTCACGT	Full length cDNA cloning of *AbMYB1*
R-cAbMYB1	GATGTAAAATTTTTTCCACGGTAT	Full length cDNA cloning of *AbMYB1*
qF-AbMYB1	CGCGGATCCATGAATTCTACTAGTAGTACT	Analysis of tissue expression profile
qR-AbMYB1	CGCGAGCTCTTAATTAAGTAGATCCCATA	Analysis of tissue expression profile
qF-AbPGK	TCGCTCTTGGAGAAGGTTGAC	Analysis of tissue expression profile
qR-AbPGK	CTTGTCGGCAATCACTACATCAG	Analysis of tissue expression profile
qF-CHS	TCGGGCTACAAGGCAAGTC	Gene expression analysis in different stages
qR-CHS	AAAAGCACACCCCAATCAAGG	Gene expression analysis in different stages
qF-CHI	AGTTGTCAATGCTCCAGTTG	Gene expression analysis in different stages
qR-CHI	GCTCTTCTTCTTCATGTTTATCG	Gene expression analysis in different stages
qF-F3H	CCTTCAACACTAACGGCTCTG	Gene expression analysis in different stages
qR-F3H	ACCTCTTCTTCCACTCTCAATATC	Gene expression analysis in different stages
qF-F3'H	ATTCACCAACCTTAGCCAAAC	Gene expression analysis in different stages
qR-F3'H	CTTAGCATCTCCCTCACAAAC	Gene expression analysis in different stages
qF-F3'5'H	CTTCTCCAATCGTCCACCTAATG	Gene expression analysis in different stages
qR-F3'5'H	CGAATGTTTGCCCAATCTTCTAAG	Gene expression analysis in different stages
qF-ANS	GGAAGAAGGAAGACTGGAGAAG	Gene expression analysis in different stages
qR-ANS	CATATTGTGGAGGATGAATGTCAG	Gene expression analysis in different stages
qF-DFR	ACTGTTGATGCTCCGATGG	Gene expression analysis in different stages
qR-DFR	ATGCTTCACCTTCTTCTTGTTC	Gene expression analysis in different stages
qF-3GT	TCCTTCTTCAACACATCACAATCC	Gene expression analysis in different stages
qR-3GT	AGTTTCCTCCTCTGCCTCTTTC	Gene expression analysis in different stages
F-AbMYB1	CGCGGATCCATGAATTCTACTAGTAGTACT	Overexpression plasmid construction
R-AbMYB1	CGCGAGCTCTTAATTAAGTAGATCCCATA	Overexpression plasmid construction
F-iAbMYB1	CGCGGTACCAAGCTTCTCATCTTCAGGCTT CATAA	RNAi plasmid construction
R-iAbMYB1	CGCCTCGAGTCTAGATTCTTCATCAAATCT CCCAACT	RNAi plasmid construction
F-35S	GACGCACAATCCCACTATCC	Identify positive transgenic plants
R-iAbMYB1	CGCCTCGAGTCTAGATTCTTCATCAAATCT CCCAACT	Identify positive transgenic plants
R-AbMYB1	5'- CGCGAGCTCTTAATTAAGTAGATCCCATA	Identify positive transgenic plants

Table S2. The genes involved in anthocyanin biosynthesis in *A. belladonna*.

gene name	gene number
AbCHS	aba_locus_13102_iso_4_len_1413_ver_
AbCHI	aba_locus_14819_iso_3_len_853_ver_2
AbF3H	aba_locus_4270_iso_5_len_1419_ver_2
AbF3'H	aba_locus_10531_iso_1_len_1673_ver_2
AbF3'5'H	aba_locus_368_iso_2_len_1707_ver_2
AbDFR	aba_locus_7485_iso_2_len_1452_ver_2
AbANS	aba_locus_408_iso_2_len_1550_ver_2
Ab3GT	aba_locus_729_iso_1_len_1448_ver_2
